# Evaluating Neonatal Oxygen Saturation From a Moderate Altitude to Below Sea Level: A Cross-Sectional Study

**DOI:** 10.7759/cureus.87787

**Published:** 2025-07-12

**Authors:** Manar Al-lawama, Razi Kitaneh, Zina Smadi, Ahmad Alhammouri, Mohammad Al-Sanouri, Farah Almudallal, Dyala Alfryjat, Lubna Al-Tarawneh, Hasan Elayan, Jaafar Darraj, Iyad Al-Ammouri

**Affiliations:** 1 Department of Pediatrics, School of Medicine, The University of Jordan, Amman, JOR; 2 Department of Psychiatry, Yale School of Medicine, Yale Univeristy, New Haven, USA; 3 School of Medicine, The University of Jordan, Amman, JOR; 4 Department of Internal Medicine, King Hussein Cancer Foundation and Center, Amman, JOR; 5 Department of Anesthesia, The University of Jordan, Amman, JOR; 6 Department of Pediatrics, The University of Jordan, Amman, JOR; 7 Department of Pediatrics, Sheikh Khalifa Medical City, Abu Dhabi, ARE; 8 Department of Pediatrics, Ghor Al Safi Hospital, Ministry of Health, Al-Karak, JOR; 9 Department of Pediatric Cardiology, The University of Jordan, Amman, JOR

**Keywords:** altitude, critical congenital heart disease screening threshold, neonate, oxygen saturation, screening guidelines

## Abstract

Background

Pulse oximetry is a vital tool for screening critical congenital heart disease (CCHD) in newborns. However, fixed thresholds may not account for physiological variations caused by altitude. This study evaluates the impact of elevation on normal neonatal oxygen saturation (SpO₂) in Jordan, spanning sites from moderate altitude to below sea level.

Methods

In a cross-sectional study, 149 healthy, full-term newborns were screened at three hospitals located at 1,050 m (Amman), sea level (Aqaba), and 420 m below sea level (Dead Sea). Preductal and postductal SpO₂ measurements were taken between 24 and 72 hours of life using standardized protocols. Statistical analyses, including analysis of variance (ANOVA) and Tukey’s honestly significant difference (HSD), were used to compare SpO₂ levels across altitudes.

Results

Mean upper-limb SpO₂ was significantly lower at 1,050 m (96.6%) compared to sea level (98.0%) and sub-sea level (97.8%) (p < 0.001). Similar trends were observed for lower-limb SpO₂. No significant differences were found between sea-level and sub-sea-level sites. Despite lower SpO₂ at moderate altitude, no newborns met the American Academy of Pediatrics (AAP) criteria for CCHD.

Conclusion

Moderate altitude results in a statistically significant, but modest, reduction in neonatal SpO₂, potentially increasing false-positive rates in CCHD screening. Our findings support the development of altitude-specific screening protocols to improve diagnostic accuracy and reduce unnecessary interventions. Further research with larger and more diverse populations is needed to inform altitude-adjusted guidelines, particularly in geographically varied regions like Jordan.

## Introduction

Early detection of critical congenital heart disease (CCHD) in newborns is essential to reducing morbidity and mortality, making timely and accurate screening a critical component of neonatal care [1]. Pulse oximetry, a non-invasive method for measuring arterial oxygen saturation (SpO₂), plays a central role in this process. It is widely regarded as a vital sign in neonatology due to its ability to detect hypoxemia, even in the absence of visible cyanosis. Pulse oximetry is used in routine newborn examinations, neonatal intensive care units (NICUs), and delivery room resuscitations, and is pivotal in screening for CCHD and diagnosing respiratory conditions [2,3].

In 2025, the American Academy of Pediatrics (AAP) updated its guidelines for screening CCHD using pulse oximetry, recommending a minimum SpO₂ of 95% in both the upper and lower limbs for a newborn to pass the screen [4]. While these fixed thresholds are broadly applicable, they do not fully account for physiological variations influenced by environmental factors - particularly altitude. The guidelines acknowledge the potential for both false-positive and false-negative results under such varying conditions. Recognizing this limitation, the AAP has emphasized the need for further studies to determine appropriate adjustments to screening protocols across different elevations, including the effects of moderate altitude on SpO₂ and screening outcomes.

It is well established that arterial SpO₂ decreases with increasing altitude [5]. High altitude (above 6,800 feet, or ~2,100 m) is known to significantly affect pulse oximetry-based screening, often resulting in lower mean oxygen saturations and a higher incidence of false positives [6,7]. Even moderate altitudes may influence these measurements, though this has been less extensively studied [8]. Some research has evaluated adaptations, such as lowering the threshold for passing or administering supplemental oxygen during screening to mitigate these effects, but further investigation is needed to determine which modifications are appropriate at different elevations [9].

In one neonatal unit included in this study, clinicians observed that some term infants experienced intermittent desaturation during the first three days of life. Despite comprehensive evaluations - including sepsis workups, cardiac echocardiography, and metabolic assessments - results were consistently normal. This led the team to question whether environmental factors, such as moderate altitude, could be delaying the physiological transition from fetal to neonatal circulation, resulting in transient hypoxemia. Subtle responses, like Valsalva-like maneuvers, were also hypothesized as potential contributors.

Motivated by these observations and the limitations of applying standard guidelines across diverse geographical settings, this study aims to investigate whether SpO₂ levels measured by pulse oximeter are influenced by moderate altitudes in Jordan. The goal is to inform context-sensitive screening strategies that ensure accurate identification of CCHD in all newborns, regardless of elevation.

## Materials and methods

Study design and setting

This cross-sectional study was conducted in three different hospitals across Jordan. The study sites included one hospital in Amman (1,050 m above sea level), a hospital in Aqaba (at sea level), and a hospital in the Dead Sea area (420 m below sea level). A convenience sample of 149 newborns was enrolled from the three hospitals included in the study.

The aim of the study is to evaluate the impact of altitude on normal SpO₂ values in newborns. The study received ethical approval from the Institutional Review Board at the Jordan University Hospital, Amman, Jordan (approval no. 2022/21575). The study was conducted in accordance with the Declaration of Helsinki and its amendments. Informed consent was obtained from the mothers after the purpose and procedures of the study were explained.

Inclusion and exclusion criteria

The inclusion criteria were: singleton, live-born, term newborns delivered at the participating hospitals; normal vital signs at birth; and a normal clinical examination performed at least one hour after feeding, to avoid hunger, excessive sleepiness, or vomiting during assessment. The exclusion criteria included: preterm birth, defined as delivery before 37 completed weeks of gestation based on the last menstrual period; diagnosis of congenital heart disease; detection of cardiac murmurs or abnormalities on cardiopulmonary examination; visible cyanosis; and clinical diagnosis of any respiratory disorder (Table 1).

**Table 1:** Inclusion and Exclusion Criteria of Study Participants

Criteria	Inclusion	Exclusion
Gestation	Singleton	Multiple
Gestational age	≤37 weeks	<37 weeks
Place of delivery	Inborn	Outborn
Prenatal diagnosis of cardiac disease	Absent	Present
Cardiac murmur	Absent	Present
Respiratory distress	Absent	Present
Cyanosis	Absent	Present
Physical examination	Normal	Abnormal

Procedure

Upon obtaining parental consent, demographic and clinical information was collected, including maternal age, gestational age, birth weight, mode of delivery, number of previous deliveries, family history of cardiac diseases, type and timing of last feeding, newborn’s exact age in hours, pregnancy complications, NICU admission, results of any prenatal scans, and maternal and newborn blood types.

A detailed cardiopulmonary examination was performed by a senior pediatric resident at each center to ensure the absence of clinical abnormalities. Each infant had a single, standardized measurement session conducted between 24 and 72 hours of life, or predischarge in cases of early discharge, in a calm, resting state following feeding. Measurements included the newborn’s temperature, respiratory rate, heart rate (in both upper and lower limbs), and SpO₂ in both upper and lower limbs, using Masimo® pulse oximetry (Masimo Corporation, Irvine, CA, USA). Pulse oximeters used across sites were factory-calibrated and subject to daily quality checks before use.

Preductal SpO₂ was measured by placing the pulse oximeter probe on the right upper limb (O2 UL), while postductal SpO₂ was measured by placing the probe on a lower limb (O2 LL). Respiratory rate was counted manually for one full minute, and heart rate was recorded from the pulse oximeter. The AAP guidelines were followed for defining the screening threshold and normal values.

Statistical analysis

All statistical analyses were performed in Python 3.10. For each altitude, we calculated the mean and standard deviation of upper limb and lower limb SpO₂. We then tested for differences across the three altitude groups using separate one-way analysis of variance (ANOVA). The null hypothesis for each ANOVA was that mean SpO₂ did not differ by altitude; significance was defined at α = 0.05. To visualize groupwise distributions and assess ANOVA assumptions of homogeneity of variance and approximate normality, boxplots of SpO₂ by altitude were generated.

## Results

A total of 149 term newborn infants were included. Of these, 86 (58%) were male infants, and 101 (68%) were born vaginally. The mean maternal age was 29 ± 6 years. Mean birth weight was 3200 ± 400 g. Table 2 provides detailed demographics.

**Table 2:** Demographic Characteristics and Saturation Values Among the Study Groups

Criteria	1050 m (50 neonates)	Sea level (49 neonates)	-420 m (50 neonates)
Male gender	28 (56%)	24 (49%)	34 (68%)
Vaginal delivery	25 (50%)	34 (69%)	42 (84%)
Mean maternal age	31 (20-42)	30 (17-43)	28 (19-45)
Mean birth weight	3,200 (2,100-4,000)	3,250 (2,500-4,400)	3,200 (2,500-4,800)
Upper limb (UL) oxygen saturation (SpO_2_%)	96.8 ± 1.2%	98.0 ± 1.3%	97.8 ± 1.2%
Lower limb (LL) oxygen saturation (SpO_2_%)	96.8 ± 1.2%	98.6 ± 1.5%	98.0 ± 1.4%

Upper‐limb SpO₂

**Figure 1:**
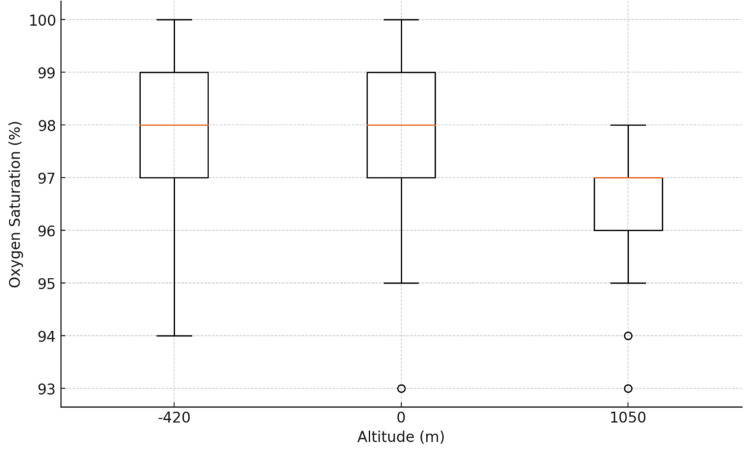
Upper Limb Oxygen Saturation by Altitude

Mean (± SD) upper-limb SpO₂ was 97.8 ± 1.2% at -420 m, 98.0 ± 1.3% at 0 m, and 96.6 ± 1.0% at 1050 m. One-way ANOVA showed a significant effect of altitude on upper-limb SpO₂ (F(2, 87) = 15.81, p < 0.0001). Post hoc comparisons (Tukey’s honestly significant difference (HSD)) indicated that values at 1050 m were significantly lower than at both -420 m (mean difference = 1.2%, p < 0.001) and 0 m (mean difference = 1.4%, p < 0.001), with no significant difference between -420 m and 0 m (Figure 1).

Lower‐limb SpO₂

**Figure 2:**
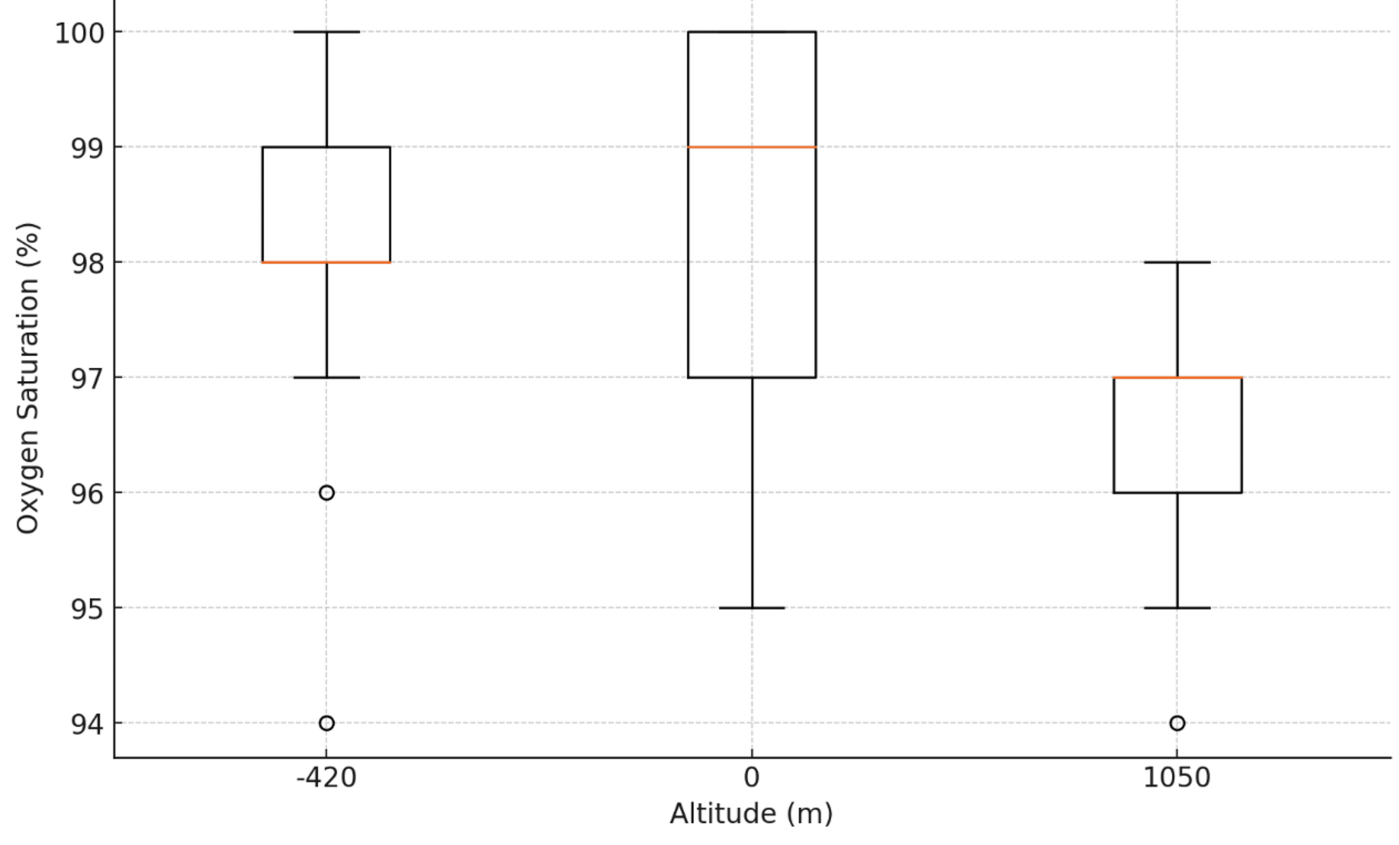
Lower Limb Oxygen Saturation by Altitude

Mean lower-limb SpO₂ was 98.0 ± 1.4% at -420 m, 98.6 ± 1.5% at 0 m, and 96.8 ± 1.2% at 1050 m. ANOVA again revealed a significant altitude effect (F(2, 87) = 32.16, p < 0.0001). Tukey’s HSD demonstrated that 1050 m measurements were significantly lower than those at -420 m (mean difference = 1.2%, p < 0.001) and 0 m (mean difference = 1.8%, p < 0.001). No difference was observed between -420 m and 0 m (Figure 2).

Boxplots (Figures 1-2) confirmed symmetric distributions with comparable variances across groups. These results establish a clear, statistically significant decline in SpO₂ at 1050 m compared to lower altitudes for both upper and lower limbs.

## Discussion

This study demonstrates that even moderate altitude can lead to a measurable reduction in peripheral SpO₂ in healthy neonates. We observed a statistically significant decline in both preductal and postductal SpO₂ at 1050 m compared to lower elevations, whereas no significant difference was noted between sea level and -420 m. These results suggest that moderate altitude influences pulse oximetry measurements, while sub-sea-level elevations do not appear to enhance peripheral oxygenation.

Such modest declines in SpO₂ with altitude align with previous research in both adults and pediatrics [10,11]. In a previous study comparing healthy term newborns at mild altitude (780 m) and sea level, Guo et al. found that SpO₂ levels were approximately 0.4% lower at altitude [12]. Rojas-Camayo et al. reported a progressive decrease in median SpO₂ across a range of altitudes in the Andes, including measurable reductions below 2,500 m [13]. In children, Binene et al. found lower reference SpO₂ values in highland regions of Papua New Guinea [14]. Similarly, Goldberg et al. noted a statistically significant decrease in arterial SpO₂ among healthy adults at 725 m compared to sea level [15]. These studies, together with our findings, reinforce that altitude-related changes in SpO₂ are evident even below the conventional “high altitude” threshold.

Although our study demonstrated a statistically significant difference in SpO₂ between moderate altitude and both sea level and sub-sea-level elevations, none of the infants screened met the AAP criteria for CCHD. It is important to acknowledge, however, that our sample size was small and included only healthy, full-term infants (≥37 weeks gestation) with normal physical examinations, which may limit the generalizability of the findings.

Nonetheless, our results, along with those of previous studies, reinforce the AAP’s concerns that standard CCHD screening protocols may yield higher false-positive rates at higher altitudes. These findings highlight the need for altitude-specific reference ranges to avoid unnecessary interventions and emphasize the importance of larger, more diverse studies to guide adjustments to screening algorithms in elevated settings.

Our study adds to this body of evidence by providing paired SpO₂ measurements from neonates at three distinct elevations, including a unique below-sea-level location. While the observed reduction in SpO₂ at 1050 m may not be clinically significant on its own, it could contribute to an increased false-positive rate when standard screening thresholds are applied. Introducing altitude-adjusted cutoffs may improve diagnostic accuracy and reduce avoidable follow-up testing.

## Conclusions

In conclusion, altitude has a measurable, dose-dependent impact on neonatal peripheral SpO₂, even at moderate elevations. These findings support the development of context-sensitive screening protocols, particularly in geographically diverse regions like Jordan. Further research involving larger cohorts is essential to refine current guidelines and ensure accurate and equitable CCHD screening for all newborns, regardless of altitude. However, our findings support the adoption of the AAP guidelines for CCHD in Jordanian hospitals and should not affect current practice. Our study provides a unique opportunity to study infants at different altitudes within the same health system. However, the small sample size, and the exclusion of neonates in the neonatal unit and preterm infants, is a limitation
